# Contrasting the value of targeted versus area-wide mosquito control scenarios to limit arbovirus transmission with human mobility patterns based on different tropical urban population centers

**DOI:** 10.1371/journal.pntd.0007479

**Published:** 2019-07-03

**Authors:** Chris M. Stone, Samantha R. Schwab, Dina M. Fonseca, Nina H. Fefferman

**Affiliations:** 1 Illinois Natural History Survey, University of Illinois at Urbana-Champaign, Champaign, IL, United Sates of America; 2 Department of Ecology and Evolutionary Biology, University of Tennessee, Knoxville, TN, United Sates of America; 3 Department of Ecology, Evolution, and Natural Resources, Rutgers University, New Brunswick, NJ, United Sates of America; 4 Center for Vector Biology, Rutgers University, New Brunswick, NJ, United Sates of America; University of Florida, UNITED STATES

## Abstract

Vector control is still our primary intervention for both prevention and mitigation of epidemics of many vector-borne diseases. Efficiently targeting control measures is important since control can involve substantial economic costs. Targeting is not always straightforward, as transmission of vector-borne diseases is affected by various types of host movement. Here we assess how taking daily commuting patterns into consideration can help improve vector control efforts. We examine three tropical urban centers (San Juan, Recife, and Jakarta) that have recently been exposed to Zika and/or dengue infections and consider whether the distribution of human populations and resulting commuting flows affects the optimal scale at which control interventions should be implemented. We developed a stochastic, spatial model and investigated four control scenarios. The scenarios differed in the spatial extent of their implementation and were: 1) a response at the level of an individual neighborhood; 2) a response targeted at a neighborhood in which infected humans were detected and the one with which it was most strongly connected by human movement; 3) a limited area-wide response where all neighborhoods within a certain radius of the focal area were included; and 4) a collective response where all participating neighborhoods implemented control. The relative effectiveness of the scenarios varied only slightly between different settings, with the number of infections averted over time increasing with the scale of implementation. This difference depended on the efficacy of control at the neighborhood level. At low levels of efficacy, the scenarios mirrored each other in infections averted. At high levels of efficacy, impact increased with the scale of the intervention. As a result, the choice between scenarios will not only be a function of the amount of effort decision-makers are willing to invest, but largely epend on the overall effectiveness of vector control approaches.

## Author summary

Control and prevention of *Aedes*-transmitted viruses, such as dengue, chikungunya, or Zika relies heavily on vector control approaches. Given the effort and cost involved in implementation of vector control, targeting of control measures is highly desirable. However, it is unclear to what extent the effectiveness of highly focal and reactive control measures depends on the commuting and movement patterns of humans. To investigate this question, we developed a model and four control scenarios that ranged from highly focal to area-wide larval control. The distribution of humans and their commuting patterns were modelled after three major tropical urban centers, San Juan, Recife, and Jakarta. We show that as implementation is applied across a wider area, a greater number of infections is averted. Critically, this only occurs if the efficacy of control at the neighborhood level is sufficiently high. A consistent outcome across the three settings was that the focal strategy was most likely to provide the best outcome at lower levels of effort, and when the efficacy of control was low. These outcomes suggest that optimal control strategies will likely have to be tailored to individual settings by decision makers and would benefit from localized cost-effectiveness modelling studies.

## Introduction

Infectious diseases continue to place a considerable burden on global human health and will likely continue to do so for the foreseeable future. Despite successes such as the eradication of smallpox and reductions in the prevalence of *falciparum* malaria, there are also many examples of pathogens with emerging or intensifying transmission or exhibiting geographic spread. Many interacting factors are involved in this dynamic, including changes in temperature and rainfall, increasing levels of urbanization, changes in land use, and an increase in commuting and travel distances [1].

These exacerbating factors lend themselves especially well to arboviruses transmitted by the anthropophilic mosquito *Aedes (Stegomyia) aegypti*, which thrives in (sub-)tropical urban settings and is often the primary vector of the dengue, chikungunya, Zika, and yellow fever viruses. Variation in dengue transmission risk, for instance, is thought to be driven largely by rainfall, temperature, urbanization and socioeconomic factors [2, 3]. Part of what makes this mosquito such an important vector in urban settings is that it undergoes its life cycle in close proximity to humans. In the larval stage, it develops in water-holding containers in or around homes, while as an adult it almost exclusively feeds on humans and can find suitable resting spots and oviposition sites in and around the domicile. Thus, with urbanization projected to rise from 54% of the world population in 2014 to 66% by 2050 [4], the amount of suitable habitat for this synanthropic vector will likely only continue to increase.

Human behavior also plays a critical role in determining the outcome of (re)emerging epidemics [5, 6]. One type of behavior that influences both the global spread of pathogens and local clustering of cases relates to human movement, both in the form of local commuting and travel or migration over longer distances. In the case of vector-borne diseases the dispersal of vectors also has to be considered in interaction with the movement across different scales by human hosts, which can shape local patterns of transmission intensity [7–9] and contribute to the heterogeneity in exposure levels typical of vector-borne disease transmission [10, 11]. In general, the implications for disease spread become complex once contacts between individuals are clustered in some manner (e.g., for spatial, household or sociological reasons) instead of assuming random or well-mixed populations [12]. In the case of vector-borne diseases, such heterogeneous patterns in exposure to infective bites can have implications for disease surveillance, estimates of risk, and control [13]. For instance, if transmission hot spots and vector population sources and sinks occur in a particular vector-borne disease system, these could potentially provide targets for control interventions [14]. Considering the movement of humans could therefore conceivably improve our ability to target both the areas of origin and the areas where onward transmission may occur [15], or at least to help reduce costs associated with those efforts.

In the case of dengue, Zika or chikungunya, such transmission clusters likely emerge from the synergy between the short distances travelled by adult *Ae*. *aegypti* [16, 17] and the multiscale movement by humans, ranging from international travel, to movement between urban centers, to day-to-day travel activities, such as commuting or going to social gatherings. For instance in Iquitos, Peru, clusters of dengue tended to be observed on greater than a 100 m radius, therefore likely involving a combination of both mosquito and human movement [18]. Other studies have supported the notion that movements within urban areas shape transmission and risk of exposure. For instance, visiting areas of risk, rather than the location of the home, was identified as a key driver of exposure to dengue in Iquitos [19]. Such day-to-day movement and travel of humans is especially pertinent for viruses transmitted by *Ae*. *aegypti* due to its diurnal biting activity, making it more likely that the place of exposure to bites is disconnected from the home environment [20]. An expected result is that a typical infected human case would lead to a number of additional human infections well outside the focal area of their home, in which control would typically be applied [19].

Understanding how different human contact structures and mixing patterns based on human movement can affect outbreak prevention and control outcomes has recently been receiving increasing attention via the use of network models. For instance, the efficacy of contact tracing has been shown to depend both on whether individuals mix assortatively or disassortatively, as well as the rate of contacts [21]. Likewise, for pathogens spread through close contact, such as influenza, vaccination strategies can potentially be optimized based on knowledge of the contact network in an area, such that strategies that make use of various aspects of this network can perform better than random immunization [22]. However, the exact role played by human movement and its impact on control strategies may vary based on the distribution and density of human populations, as well as socioeconomics or culture. For instance, commuting flows within urban centers can differ drastically between cities, based on their size and distributions of residential and work places. The resulting differences in mobility patterns can likewise affect epidemic spread [23, 24].

For *Aedes*-transmitted viruses, especially dengue, examples of successful use of vector control interventions to reduce exposure risk certainly exist, such as a successful larval source management and public education strategy employed for a significant period in Singapore [25]. However, application of vector control interventions often fails to prevent arboviral outbreaks, possibly due to inadequate control responses [26, 27]. The scope and level of response, as well as how long the response is sustained, are all recognized as critical factors that determine the overall success of implementation. Improving implementation strategies is thus critical to improving *Aedes*-borne virus control programs [27]. Given the importance of human movement and the focal nature of transmission, it is tempting to consider control strategies that explicitly make use of knowledge of human mobility patterns. For instance, in a previous study, we found that the relative efficacies of area-wide versus more targeted control responses in limiting outbreak size depended on both the daily rate of commuting and the proportion of patches that implemented control interventions [28]. At the same time, while strategies at the household level (e.g., a form of contact tracing whereby households connected to positive cases are treated) may be appealing, contact tracing in urban tropical centers in developing countries may be logistically infeasible [19]. Here, we explore a number of straightforward vector control implementation scenarios using a simplified spatial model of Zika transmission by *Ae*. *aegypti*. Despite their simplifications, patch-based models can nonetheless provide important insights (due to their more tracteable nature) into questions related to the role of commuting and transmission and optimal control (e.g., [24, 29]). The simplifications we use here relate largely to the scale of movement and targeting of control within the model (i.e., we use a relatively large patch size, namely at the level of the neighborhood, within which we assume humans are well-mixed). This model is used to develop general insights into when and how human movement and the distribution of humans over the urban landscape should affect decisions regarding the scale of interventions. We explicitly do not intend for this model to be predictive or provide advice to local vector control decision-makers, and would recommend household- or individual-level models specifically parameterized to local conditions for that. Here, we explore the relative efficacy and effort involved in vector control implementation under the following scenarios: (1) focal (individual neighborhood) control implementation; (2) two types of area-wide control; and (3) control implementation in both an individual neighborhood and in the neighborhood most strongly connected to it via *a priori* knowledge of human movement patterns. We specifically investigate whether the derived strategic insights would be consistent among tropical urban centers of varying sizes, layouts, human distributions, and movement patterns, or whether strategies would have to be tailored to individual locations. To do so, we ran extensive sets of simulations for models coarsely capturing the human distributions and movement patterns of San Juan (Puerto Rico), Recife (Brazil), and Jakarta (Indonesia)—all locations where *Ae*. *aegypti* has or could potentially lead to Zika epidemics.

## Methods

### Model description

We developed a discrete, stochastic version of a spatial compartmental vector-borne pathogen transmission model, which was previously used to explore spatial aspects of mosquito control on a simplified grid-based landscape [28]. Here, in this metapopulation model individual patches represent neighborhoods or districts. Briefly, the model is suitable for microparasites where within-host dynamics can safely be ignored and infection status can thus be modelled as a population-level characteristic. We assume a pathogen with a single host species and a single vector species, which is a reasonable assumption for arboviruses such as dengue, chikungunya, or Zika viruses, transmitted by *Aedes aegypti* in many urban tropical areas [30]. The model parameterization is based on Zika virus (S1 Table).

The human (host) and mosquito (vector) populations are tracked by infection status and life stage. Within each patch (neighborhood or district of a particular location, indicated by subscript *k*), the human population (*N*_*h*,*k*_) consists of susceptible (*S*_*h*,*k*_), exposed or latent (*E*_*h*,*k*_), infectious (*I*_*h*,*k*_), and recovered or immune (*R*_*h*,*k*_) hosts. The vector population (*N*_*v*,*k*_) is made up of immature (*L*_*v*,*k*_), susceptible (*S*_*v*,*k*_), exposed (*E*_*v*,*k*_) and infectious (*I*_*v*,*k*_) mosquitoes. See the supplementary material for the equations that describe the transitions between these compartments.

**Fig 1:**
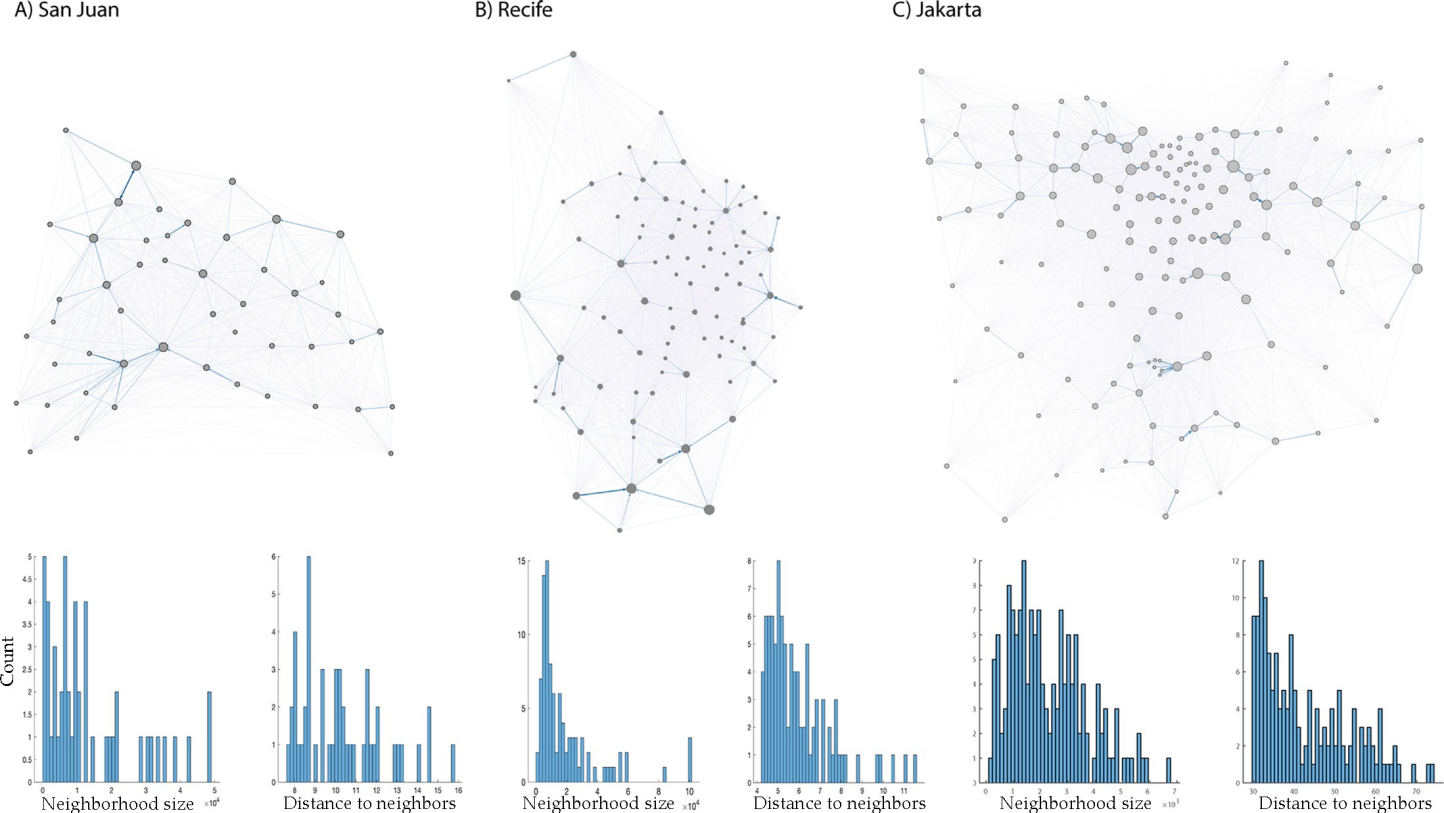
**An overview of the spatial distribution of patches (located at the centroid of their respective neighborhoods), their relative size, and the connectivity between neighborhoods based on a gravity model for San Juan (A), Recife (B), and Jakarta (C).** Histograms show the distributions of population sizes per neighbordhood and the mean distance to other neighborhoods per neighborhood, for the three cities.

We base the spatial configuration of the model on three areas where dengue and/or Zika virus epidemics have been known to occur in recent years. The purpose is simply to have a diversity of spatial distributions of neighborhoods in tropical urban settings. The cities which informed our spatial modelling were San Juan (Puerto Rico), Recife (Brazil), and Jakarta (Indonesia), though other differences between those cities (e.g., rainfall patterns or seasonality) were not considered. A figure with directed graphs and information on sizes and relative distances between patches is provided (Fig 1). We thus also kept entomological details as simple as possible, and adjusted the density-dependent rate of larval mortality for each neighborhood such that the vector:host ratio was similar in all neighborhoods and across settings (ca. 5 female mosquitoes per human). We used the lowest available level of administrative boundary for each city (i.e., neighborhoods or districts) to inform a model of human movement, as well as the structuring of vector control (i.e., we assume the most intense form of targeting occurs at the scale of one patch or neighborhood and ignore the role of movement or heterogeneity within patches). The choice of neighborhoods as our smallest spatial unit was driven by two reasons: we wanted to cover areas that were large enough so that we could ignore the movement of mosquitoes, and to keep the model simulations from becoming computationally too demanding. It is likely true that within these neighborhoods human populations are not homogeneously distributed or exposed to mosquito bites. To capture such heterogeneity (which would even occur within households), one would need an individual-based approach with much finer granularity. As we were interested in the broad question of whether the distribution of humans over the landscape and their movement might affect vector control strategies, and the patch size has little effect on that question, we use this simpler model to derive strategic insights (but would suggest using individual-based models that account for local spatial and temporal distributions of vectors, vector control measures that are already in place, and other forms of local parameterization, for predictive purposes and policy recommendations).

We assume mosquito dispersal is sufficiently limited between neighborhoods relative to the movement of hosts that we can ignore the impact of mosquito movement. The size of the human population was derived from gridded data from the WorldPop project (www.worldpop.org) [31, 32] and summed over the districts. Human movement reflected commuting, such that a proportion of the population of each neighborhood would spend time at another patch each day. Specifically, each host has a specific home patch (*k*), but can potentially be exposed to infective bites in other patches (*j*) with a probability of *δ*. The distribution of hosts that commute to other neighborhoods depends on both the distance to and population size of the neighborhoods, following a gravity-type model [33].

$$F_{j,k}=\frac{\left|j\right|}{\left|k\right|+\left|j\right|}\frac{\left|k\right|\;\left|j\right|}{d_{j,k}^{2}}$$ (1)

Where |*j*| and |*k*| are the respective number of humans residing in neighborhoods *j* and *k*, and *d*_*j*,*k*_ represents the distance between these neighborhoods. Distances between neighborhoods were calculated by taking the centroid of each neighborhood and using the “distm” function of the geosphere package in R [34]. These probabilities (of commuting from patch *k* to each other patch *j*) are then normalized over all neighborhoods. For each patch, we then have a matrix *W* with probabilities of remaining in the home patch (with probability *w_k,k_* = 1−*δ*, or to any other patch with probability $w_{j,k}=\delta F_{j,k}^{*}$. The distribution of hosts per day over patches is based on draws from a multinomial distribution with these probabilities.

### Control scenarios

We explored four distinct control scenarios where the presence of infected humans in a given patch triggered responses that varied from highly focal to collective and area-wide. Specifically, we had 1) a focal or individual-patch level response; 2) a targeted response, in which control occurs in the patch with infections as well as the patch with which it is most strongly connected; 3) a limited area-wide response, wherein control occurs in all neighborhoods within a certain radius (0.33 x the mean distance among all neighborhoods in that locality–a value chosen to lead to a scenario intermediate between scenarios 1 and 4) of the patch with infective humans; and 4) a collective, area-wide response, wherein all neighborhoods initiate control. We also obtained baseline estimates of the progression of the epidemic in the absence of mosquito control.

After a threshold of 2 infected humans is detected in a participating patch (as many infections will be asymptomatic [35], we assume detection occurs only for a proportion of infections, based on draws from a binomial probability distribution with probability of success of 0.5), control is initiated after 1–30 days (randomly chosen for each simulation) to reflect a time delay associated with factors such as laboratory processing of samples, the incubation period of the virus, and health-seeking behavior of symptomatic individuals. To focus on the impact of the spatial configuration and scale of control, we used the same control method in each scenario. Specifically, we assumed larval control (e.g., the application of pesticides to larval development sites) was used, modifying the base rate of death of larvae as follows: $\mu_{c}=-\log(1-\frac{\theta }{100})+\mu_{2}$, where θ is the effectiveness of the control method or the percentage of immature mosquitoes in a given patch that are killed per day due to the insecticide. The level of induced mortality was chosen randomly from values ranging from 1–100% (drawn from a uniform distribution).

### Analysis

For each scenario in each of the three localities, we performed sets of 2000 simulations. Each simulation had a 50-day burn-in period to allow mosquito population sizes to reach stable values, after which a single infected human was introduced to a randomly chosen patch. The infection dynamics were then followed for a two-year period, to capture both the major epidemic peak after initial (re)emergence and a significant stretch of time afterwards to allow the system to stabilize beyond transient dynamics. To gain insight into the usefulness of various control strategies, we compare the number of infected and recovered humans at the end of the two-year period for each of the 4 intervention scenarios to the scenario without any control, based on simulations where the initial introduction spread. We also investigated how the dynamics of control depend on assumptions regarding the efficacy of the control effort and the location of neighborhoods participating in the control effort by performing additional sets of simulations where a randomly chosen 20% of neighborhoods were assumed to not participate. For these particular sets of simulations, we assumed efficacy of larval control per patch was 60%.

As a measure of the level of investment or effort associated with each scenario, we kept track of the total number of persons covered (number of inhabitants per patch x number of days that patch initiated control, summed over all patches). Although assessing the financial and economic costs associated with the different larval control strategies were beyond the scope of this analysis (and we therefore likewise ignore complications such as economies of scale), we draw upon health economic methods to estimate the net benefits of each of the four control strategies, using the ‘no control’ scenario as our reference case. We calculated the net benefit of each simulation as the number of infections averted multiplied by an investment threshold value, which here represents the number of persons a city or control program is willing to cover in order to avert one infected case, minus the number of persons covered in the control scenario [36]. We then used the R package BCEA [37] to graph the probability (i.e., the proportion over all simulations in which a given scenario had the greatest net benefit) that each scenario was most effective for a given amount of effort across a range of investment thresholds.

## Results

**Fig 2:**
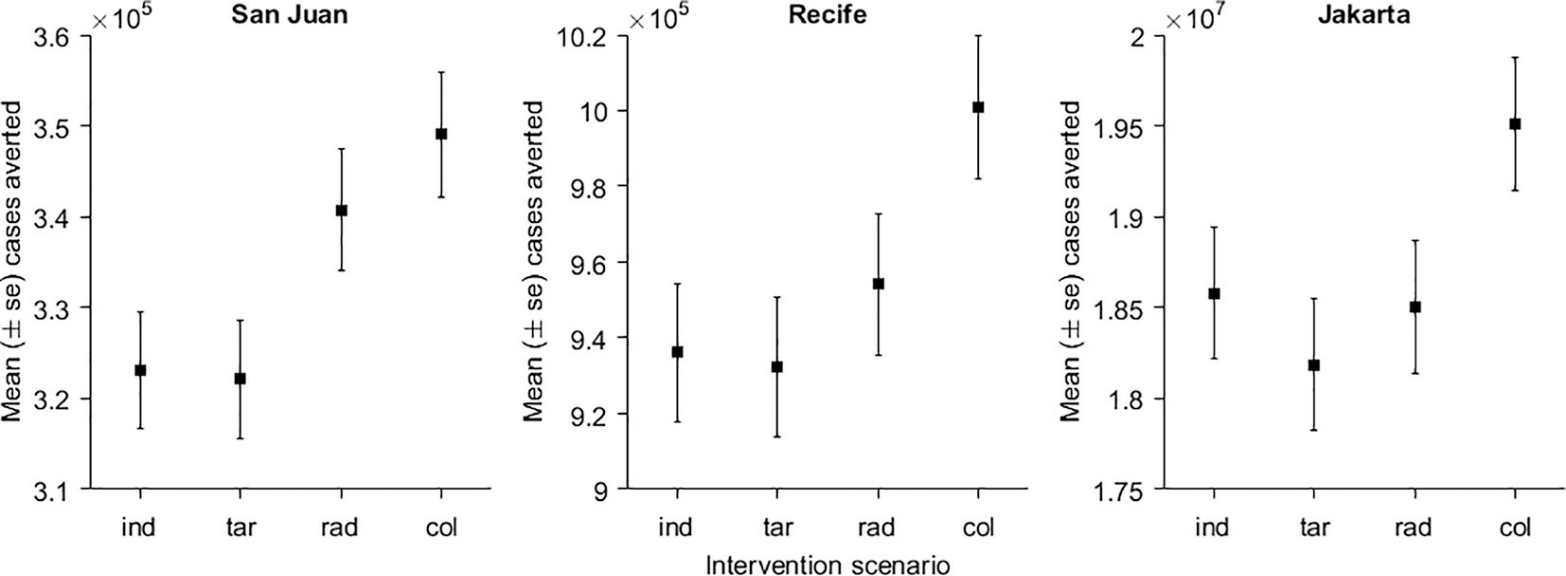
Infections averted by the end of the two-year period for the individual (ind), target (tar), radius (rad), and collective (col) strategies in host commuter networks modelled after San Juan, Recife, or Jakarta.

In general, all four control scenarios substantially limited the severity of the outbreak, with the collective response in particular having a greater impact. In terms of infections averted over the two-year period, compared to the scenario without larval control, the focal and targeted responses were consistently the least effective. The relative impact of the intermediate area-wide scenario depended on the urban center (e.g., its size, the number of neighborhoods and the distribution of humans across the locality) in which the epidemic took place (Fig 2). In San Juan, the increase in effort associated with the two area-wide interventions resulted in a concomitant increase in efficacy. In Recife, the focal and targeted response were only slightly worse than the intermediate area-wide response in terms of impact, while the collective response was significantly more effective. In contrast, in Jakarta, the three limited responses were much closer to each other in terms of impact.

**Fig 3:**
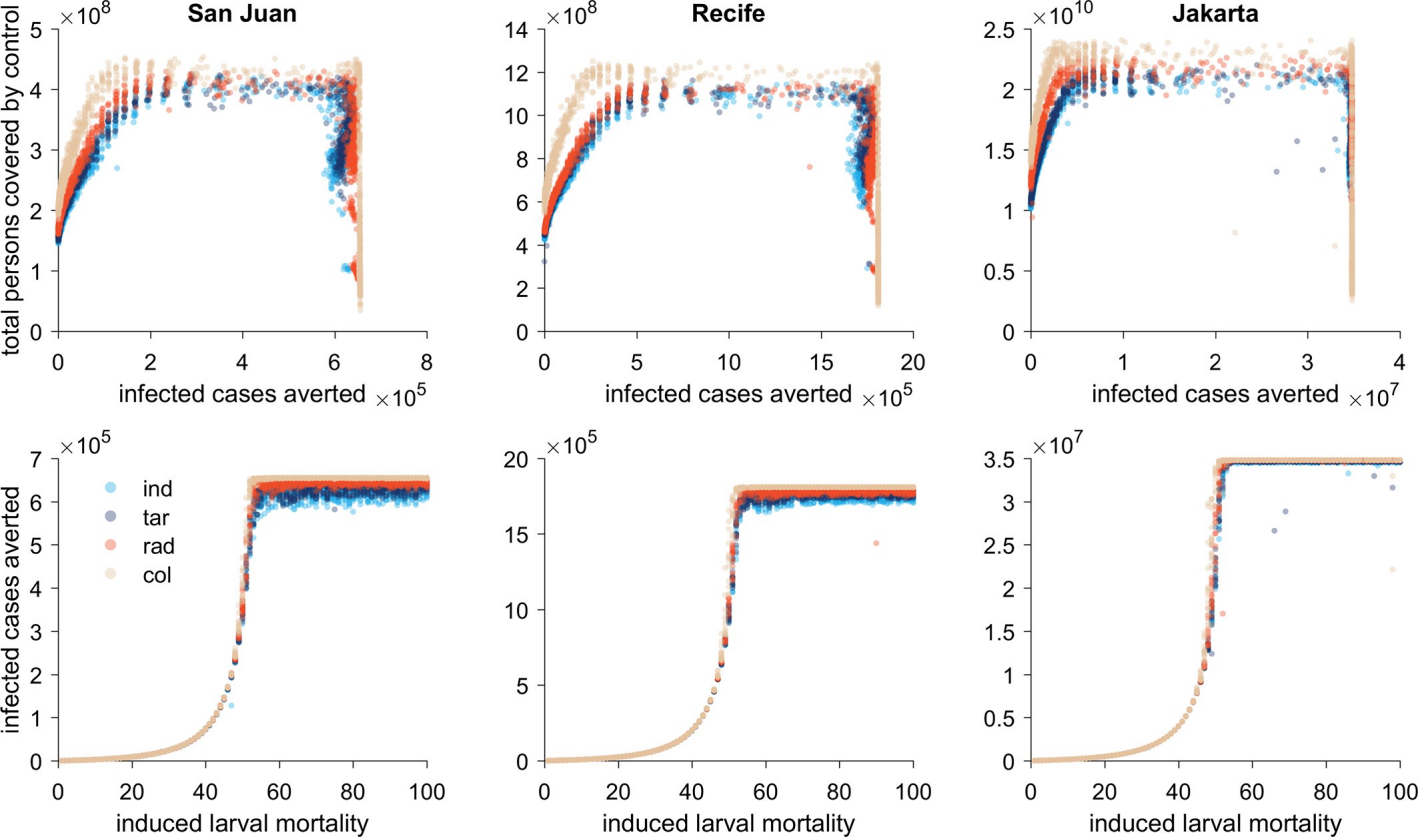
Top: Scatter plots of the number of infections averted per iteration plotted against the total number of people covered in that simulation run for the four intervention scenarios (individual, targeted, radius, collective) and three settings (San Juan, Recife, Jakarta). Bottom panels: Scatter plots of the number of infections averted (compared to the scenario without interventions) in relation to the level of induced larval mortality (i.e., efficacy of the larval control at the neighborhood level) for the four scenarios and three settings.

**Fig 4:**
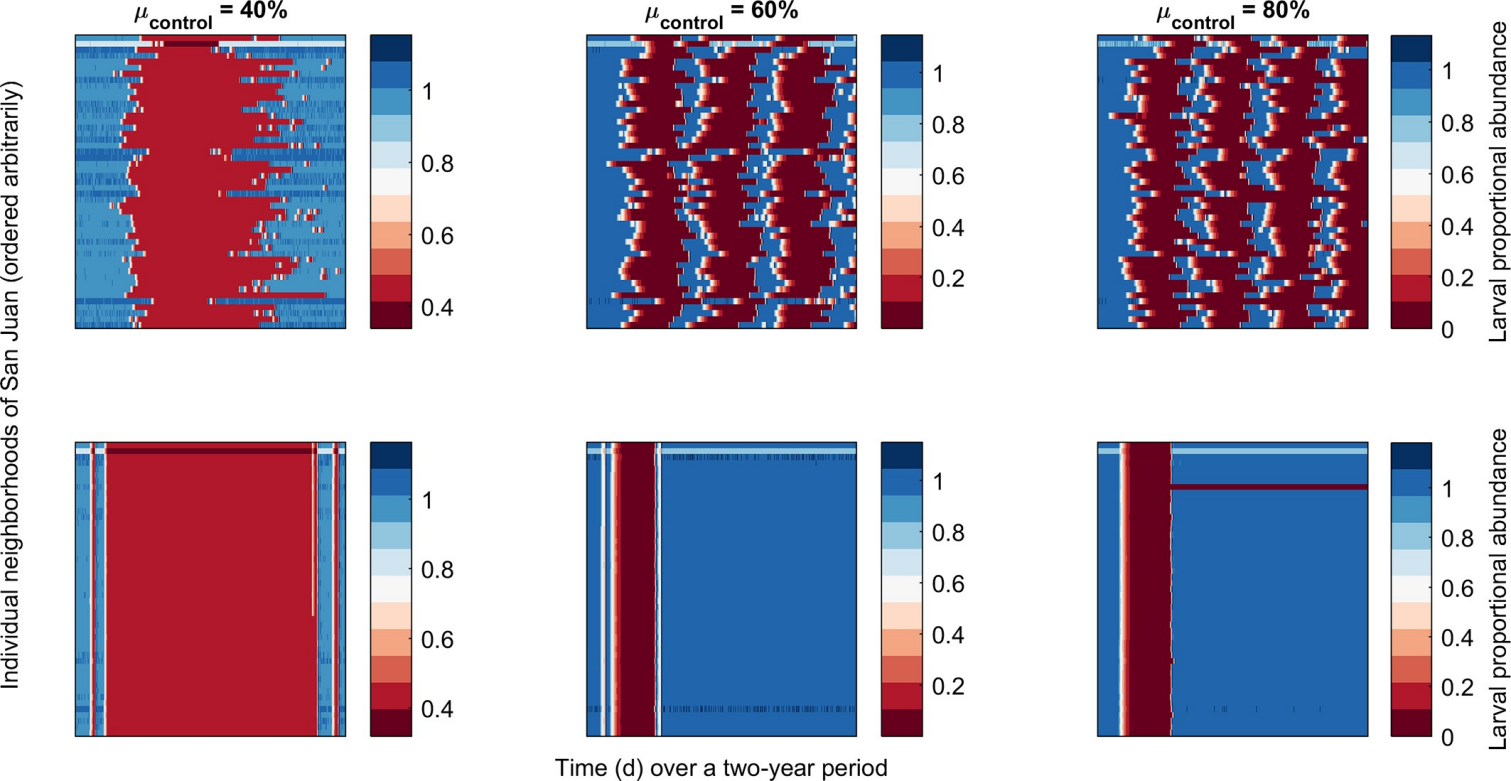
Heatmaps of larval populations in 49 districts (y-axis) over time (x-axis), for San Juan. Darker red areas reflect low levels of larval populations due to control. Top row is for an individual, focal strategy, the bottom row for the collective strategy. Efficacy of larval control (the proportion of larvae killed by larval control per day) was 40% (left), 60% (center), or 80% (right).

An exploration of individual simulations via scatter plots of infections averted versus the total number of people covered by larval control over the two-year period reveals the complex relationship between investment and efficacy (Fig 3, top panel). There are two distinct patterns visible in these scatterplots: an area where the four strategies differ largely in the extent of the number of persons covered, but with no clear improvement in outcome; and an area where scenarios that cover more persons also result in a greater number of infections averted. A similar distinction is evident when looking at the number of infections averted per simulation in relation to the assumption of efficacy of larval control at the patch-level (i.e., the induced level of larval mortality per day in a patch that enacts larval control)—at lower levels of efficacy there is no discernable difference in outcomes between the interventions, while at higher levels the more collective responses do achieve a greater impact (Fig 3, lower panels). A possible explanation for this phenomenon is as follows (Fig 4): when the efficacy of larval control is low (e.g., 40%), this intervention is unable to contain the spread of the infection and regardless of the response type, control is triggered everywhere. In other words, the focal response and collective response become essentially equivalent. This is in contrast to control at higher levels of efficacy, where we see that under a collective response, control ceases when transmission is interrupted. Under the focal control strategy, however, the virus maintains itself in the metapopulation triggering waves of control throughout the two-year period.

**Fig 5:**
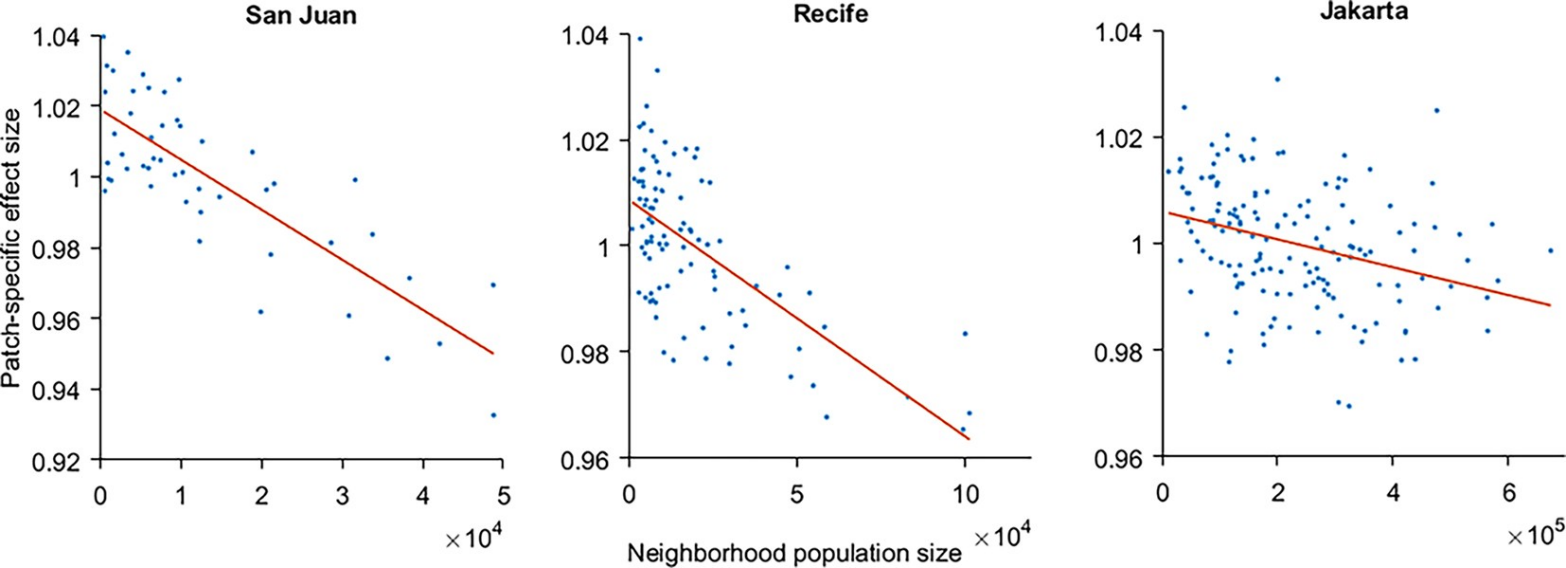
The relative impact of including a particular neighborhood in the intervention response (mean prevalence of simulations in which a particular neighborhood was included divided by the mean prevalence over all simulations) in relation to the population size of that neighborhood, based on the individual response scenario. *R*^*2*^ values are 0.67, 0.56, and 0.11 for San Juan, Recife and Jakarta, respectively.

We additionally performed simulations where we assumed that control would not be feasible in a proportion of randomly chosen and varied neighborhoods (20%). Based on this we can investigate what the relative impact is of including or excluding specific neighborhoods from the control responses. Our results suggest (and support findings from a different model and scenarios [38] that the spatial configuration of control also impacts the effectiveness of the control response (S1 Fig). This is likely due to the connectivity structures of the patches (derived from a gravity model). In part, this also appears to relate to the population size of each specific neighborhood (Fig 5). However, this result was most evident for San Juan (*R*^*2*^ = 0.67) and was weaker in both Recife (*R*^*2*^ = 0.56) and particularly Jakarta (*R*^*2*^ = 0.11). In these larger environments, other factors (e.g., distribution of patches) likely become more prominent.

**Fig 6:**
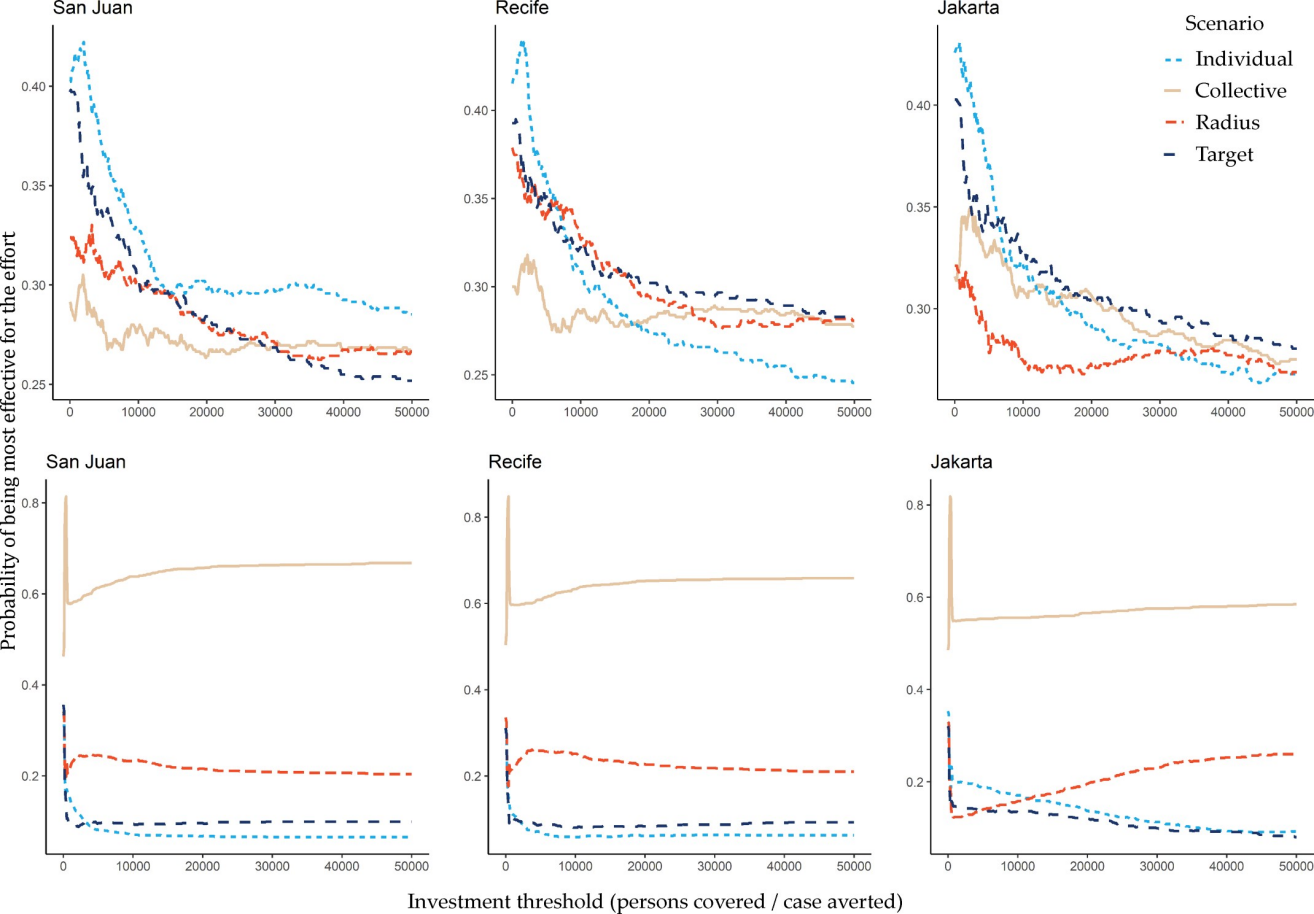
The probability that a particular intervention scenario provides the greatest net benefits (i.e., the probability for each of the different scenarios that they are most effective at that level of effort) along a range of investment thresholds (the number of persons that have to be covered by larval control in order to avert a single case), by location. A scenario without larval control was used as the reference case. The upper panel shows outcomes when the efficacy of larval control was low (<50%), while the lower panel shows the outcomes for simulations where efficacy was high (≥50%).

All three settings displayed similar relationships between the level of investment (number of persons covered per infected case averted) and the net benefits of control. (Fig 6). We analyzed these relationships separately for low (<50%) and high (≥50%) levels of efficacy of larval control. There is a striking difference in which interventions provide the best value. At high levels of efficacy, despite having to cover all patches at once in response to a trigger, the collective response is most likely to provide the best outcome for a given level of effort. When control is less efficacious, the results are more variable between the sites. At low investment thresholds, the focal response provides the best outcome. In San Juan, the focal response is the most effective for any level of investment threshold, while in the other locations the radius, targeted, and collective approaches become more appealing at higher levels of investment.

## Discussion

An important question in vector-borne disease control is how to structure prevention and/or control interventions optimally in space and time. This is particularly relevant for *Aedes*-transmitted arboviruses such as Zika, dengue, and chikungunya, which tend to be highly focal both temporally and spatially [39, 40]. The fact that a large proportion of infected humans (at least in the case of dengue) remains asymptomatic yet contributes to ongoing transmission only complicates effective focal test and control strategies for such pathogens [41]. Given the tremendous burden on human health associated with *Aedes*-transmitted viruses, and the fact that the majority of this burden is experienced in tropical, often resource-constrained settings, there is a clear need for insight into what would constitute the most effective control strategy for a given amount of investment of effort. An example of this tension is provided by Liebman et al [18], who suggest that while the current World Health Organization (WHO) guidelines recommend implementing vector control in a 400 m radius around a detected human case [42], control responses in Iquitos, Peru tended to be limited to a radius of 100 m, as a 400 m radius could involve treating hundreds of houses, overly straining time and resources. Yet even the 400 m boundary suggested for perifocal responses would not limit transmission if responses are not immediate or the case in question has travelled outside the targeted area [42]. In other words, control responses to outbreaks could be vastly improved by taking the extent of movement into account. Here, we explored this topic using coarse models of human populations and their movement modelled after three different urban tropical centers by investigating the relative impact of different control implementation strategies that ranged from focal to collective, area-wide approaches.

Our results illustrate that, as might be expected, collective (city-wide) responses are considerably more effective at limiting total outbreak size than are the more focal strategies. In general, the number of infections averted (compared to a control scenario without any intervention) increased with increasingly ambitious strategies. However, the relative impact of the two intermediate strategies (treatment based on a radius around the focal neighborhood, and treatment in both the focal and the most strongly connected neighborhood) differed between the three cities. The targeted approach in particular appeared to offer no benefit over the focal response.

The reasons for these differences are not immediately obvious but could be tied to differences in scale (population, physical size, number of neighborhoods, etc.). This suggests that optimizing control responses will have to be done on a location-by-location specific manner. Critically, however, when we estimated the effort involved in enacting each of the control scenarios (by summing the total number of persons covered over all days of treatment) and used this metric to investigate which scenarios were the most effective for a given amount of effort, the outcomes are remarkably consistent between the urban centers. When efficacy of control is high, the collective response provides the best value. This is likely because although it is costly to treat all locations simultaneously, this strategy then can succeed at quickly interrupting transmission of the virus. Two important caveats are that we only introduce the pathogen at a single time point, and that this high level of efficacy of larval control is unlikely to be achieved in reality due to the cryptic and abundant larval habitats employed by *Ae*. *aegypti*. For lower levels of efficacy, the scenario that offers the best value varies by investment threshold and location. At lower investment thresholds, the focal scenario is likeliest to provide the best value, while at high levels, the targeted response provides the best value in Recife and Jakarta, while in San Juan the focal response is consistently the most effective for a given level of effort. Whether such insights would still be evident were we to extend this modelling approach with appropriate costing and economic models was beyond the scope of the current study, but would be required before making policy recommendations.

We also investigated whether there might be differences in the most effective spatial configuration of control, and discovered that there were certain neighborhoods which, if included in the control scenario, tended to increase or decrease the overall effectiveness. The neighborhoods whose inclusion most increased effectiveness tended to have lower human population sizes in San Juan, though this relation was somewhat (in Recife) and considerably (Jakarta) weaker in the other two environments. Because we used a constant ratio of vectors to hosts, this outcome does not relate to differences in vectorial capacity directly. As our movement was informed by a gravity model, smaller neighborhoods will receive fewer commuters. Larger neighborhoods, on the other hand, will attract visitors from a larger number of patches and therefore be better mixed, which can lower the intensity of transmission. This would suggest that in certain areas it may be worthwhile to target less well-mixed neighborhoods. It is not clear why the impact of neighborhood size on effect size decreased in the larger cities. It is possible that above a certain scale, factors related to the spatial distribution or configuration of patch sizes becomes more relevant. The criteria for selecting specific neighborhoods for control may in that case have to take the scale of urban centers into account.

There were a number of simplifying assumptions of the model and simulations. For instance, human movement as simulated here is coarse and captures only commuting behavior at a between-neighborhood level, rather than fine-grained distinctions that in reality occur between the distances travelled, time spent at different locations, and frequency at which particular types of habitats (e.g., residential, recreational, commercial, etc.) are visited [43, 44]. Similarly, we assumed that the movement patterns could be well-described with a gravity model, and for some settings, other models of movement may capture reality better (e.g., radiation models [45]). In other words, the current study represents an important first step at investigating how the level of coordination between different neighborhoods may vary for different spatial compositions of humans, but it is certainly not an exhaustive exploration of the implications of human movement patterns for disease control. One particularly interesting complication relates to the effect of sickness behavior of febrile cases on mobility patterns. In the case of dengue, it has been qualitatively shown that individuals presenting with fever and testing positive for dengue visited fewer locations and spent more time inside their home than afebrile study participants [46]. It is likely that such effects will vary over the course of infection and depend on the severity (or lack) of symptoms. Including such sickness-mediated changes in behavior in a transmission and control model as used here was beyond the scope of the current study, but would be important to consider as both the transmission dynamics and implications for control strategies could change drastically. Another assumption we made relates to the likelihood of infections triggering control. We based this on two humans, and assumed the probability of detecting these infections was informed by an estimate of an symptomatic:asymptomatic ratio of 1:1 (e.g., [35]). This may still be an overestimate of the probability of detecting an infection, either due to health-seeking behavior or inefficiencies in health care systems. We additionally assumed control would only be triggered upon detection of two infected humans, whereas a single case may be used in reality (e.g., [47]). These respective over- and underestimates of triggering events will cancel each other out to an extent. The implications of varying the trigger for Zika control has been investigated recently [48].

Although costs are not captured here in an economic or financial sense, we do provide a crude measure of effort by looking at the total number of persons that would be covered by control efforts. This, therefore, disallows comparisons on a monetary scale, and also necessarily ignores a variety of complications (e.g., future discounting, (dis)economies of scale [49]) that are all necessary to consider in actual cost-effectiveness studies. Thus, although our approach provides general insight into the relative efficacy of the various strategies, it would be necessary to consider actual costs associated with the various scenarios in future studies.

To conclude, we have investigated both how the spatial scale of control strategies and the choice of neighborhoods which are included in interventions may influence the efficacy of control in limiting outbreak size. Our results suggest that the overall best choice of strategy will largely depend on decision-makers’ willingness and ability to invest in control measures (which is likely to be location specific and may even vary within cities, depending on the organization of vector control), and the efficacy of control methods. The spatial configuration or distribution of control (i.e., the neighborhoods whose inclusion in scenarios were likely to increase the effectiveness of the scenario) appeared to differ by urban center, where for San Juan (a relatively smaller city) and to a somewhat smaller extent Recife, the inclusion of neighborhoods was at least in part driven by the human population size, whereas targeting based on this factor became less important in Jakarta. While this finding needs further investigation, it adds to the conclusion that there will be no one-size-fits-all optimal solution for vector control strategies between different environments. Further work is needed to determine how such strategies should be structured, and to what extent that will vary between different urban centers of different sizes, distributions, and movement patterns of humans, under different frequencies of introductions of pathogens, and tailored to individual environmental, seasonal, and entomological settings.

## Supporting information

S1 Text Model equations. Description and equations of the stage transitions and force of infection in the model.
(DOCX)

S1 Table Description of parameters. (DOCX)

S1 Fig **Individual neighborhoods in the San Juan area (top row), Recife (middle row) and Jakarta (bottom row), with total population size of humans per neighborhood (left) and the relative impact or effect size of including a particular neighborhood in the control response (i.e., mean prevalence of simulations in which a particular neighborhood was included divided by the mean prevalence over all simulations).** The latter is based on the individual response scenario.
(TIF)
