# Supplementary material for: Contrasting the value of targeted versus area-wide mosquito control scenarios to limit arbovirus transmission with human mobility patterns based on different tropical urban population centers
Source: PLoS Negl Trop Dis. 2019 Jul 3;13(7):e0007479. doi: 10.1371/journal.pntd.0007479 (PMC6608929; doi:10.1371/journal.pntd.0007479)
Supplement: S1 Text — Description and equations of the stage transitions and force of infection in the model. (DOCX) [file pntd.0007479.s001.docx]

**Supplementary Material**

*Model description*

*Stage transitions:*

A description of the parameters and their values is provided in Table S1. The transitions between compartments are governed by the following set of equations:

$$S_{h,k}\left(t+1\right)\text{ }=\text{ }S_{h,k}\left(t\right)\text{ }-\text{ }p_{1}$$

where $p_{1}\sim Poisson(\sum_{j} w_{j,k}S_{h,k}(t)\lambda_{h}(j,t))$, the number of susceptible hosts from patch *k* that become infected at time *t* (see below).

$$E_{h,k}(t+1)=E_{h,k}(t)+p_{1}-p_{2}$$

where $p_{2}\sim Poisson(\tau_{h}E_{h,k}(t))$ gives the number of latent hosts that progress to the infective state.

$$I_{h,k}(t+1)=I_{h,k}(t)+p_{2}-p_{3}$$

where $p_{3}\sim Poisson(\gamma I_{h,k}(t))$ gives the number of infectious hosts that progress to the recovered or immune state.

$$R_{h,k}(t+1)=R_{h,k}(t)+p_{3}$$

The focus of the model is on the short-term, so that human demography and any possible waning of immunity can be ignored. Vector populations are described as follows:

$$L_{v,k}(t+1)=L_{v,k}(t)-p_{4}+p_{5}-p_{6}$$

Here the number of immature mosquitoes dying per time step is given by $p_{4}\sim Poisson(\mu_{2}+(\mu_{3}L_{v,k}(t))L_{v,k}(t))$. The number of immature mosquitoes added each step are given by . Juvenile development is given by $p_{6}\sim Poisson(\eta(L_{v,k}(t)-p_{4}))$.

Changes in the population size of susceptible (uninfected) mosquitoes are given by:

$$S_{v,k}(t+1)=S_{v,k}(t)+p_{6}-(p_{7}+p_{8})$$

where losses are due to mortality: $p_{7}\sim Poisson(\mu_{1}S_{v,k}(t))$, and due to infection: $p_{8}\sim Poisson(\lambda_{v}(k,t)(S_{v,k}(t)-p_{7}))$.

$$E_{v,k}(t+1)=E_{v,k}(t)+p_{8}-(p_{9}+p_{10})$$

Mosquitoes leave the exposed class by dying: $p_{9}\sim Poisson(\mu_{1}E_{v,k}(t))$, and by becoming infectious following the extrinsic incubation period: $p_{10}\sim Poisson(\tau_{v}(E_{v,k}(t)-p_{9}))$.

$$I_{v,k}(t+1)=I_{v,k}(t)+p_{10}-p_{11}$$

Infectious mosquitoes remain so until they die: $p_{11}\sim Poisson(\mu_{1}I_{v,k}(t))$.

*Force of infection*

The forces of infection on hosts (λ_h_) and vectors (λ_v_) are:

$$\lambda_{v}(j,t)=ac\frac{\hat{I}_{j}(t)}{\hat{N}_{j}(t)}$$

where $\hat{I}_{j}(t)=\sum_{k} w_{j,k}I_{h,k}$ and $\hat{N}_{j}(t)=\sum_{k} w_{j,k}N_{h,k}$. In other words, the force of infection on vectors in a given patch depends on the biting rate (*a*), the probability of a vector becoming infected when biting an infective person (*c*), and the proportion of hosts present in patch *j* that are infective.

The force of infection on hosts, λ_h_, is given by:

$$\lambda_{h}(j,t)=ab\frac{I_{v,j}(t)}{\hat{N}_{j}(t)}$$

That is, the force of infection on humans present in patch *j* depends on the biting rate (*a*), the probability of a human becoming infected following an infective bite (*b*), and the density of infective vectors in that patch over all hosts that are present there that day.
