# Supplementary material for: Contrasting the value of targeted versus area-wide mosquito control scenarios to limit arbovirus transmission with human mobility patterns based on different tropical urban population centers
Source: PLoS Negl Trop Dis. 2019 Jul 3;13(7):e0007479. doi: 10.1371/journal.pntd.0007479 (PMC6608929; doi:10.1371/journal.pntd.0007479)
Supplement: S1 Table — (DOCX) [file pntd.0007479.s002.docx]

**Table S1: Description of parameters**

| **Parameter** | **Description** | **Value** | **Dim.** | **Source** |
| --- | --- | --- | --- | --- |
| δ | proportion of exposure experienced away from home patch | 0.4 | d^-1^ | - |
| a | inverse of gonotrophic cycle duration | 0.5 | d^-1^ | - |
| b | probability of a host becoming infected upon receiving an infectious bite | 0.35 | - | [1] |
| c | probability of a vector becoming infected upon biting an infective host | 0.31 | - | [1] |
| τ_h_ | inverse of human latent period | 1/5.8 | d^-1^ | [2] |
| γ | inverse of human infectious period | 1/5.8 | d^-1^ | [2] |
| τ_v_ | inverse of extrinsic incubation period | 1/9.1 | d^-1^ | [2] |
| µ_1_ | adult mosquito death rate | 1/13 | d^-1^ | [3] |
| µ_2_ | base immature mosquito death rate | 0.05 | d^-1^ | - |
| µ_3_ | density-dependent modifier of immature mortality | 0.0000005-0.00000003 | d^-1^ | varied |
| φ | fecundity | 10 | d^-1^ | [4] |
| η | rate of larval development | 1/7 | d^-1^ | [5] |

**References**

1. Manore C, Ostfeld R, Agusto F, Gaff H, Ladeau S. Defining the risk of Zika and chikungunya virus transmission in human population centers of the eastern United States. PLoS Negl Trop Dis. 2017;11:e0005255.

2. Funk S, Kucharski A, Camacho A, Eggo R, Yakob L, Edmunds W. Comparative analysis of dengue and Zika outbreaks reveals differences by setting and virus. PLoS Negl Trop Dis. 2016;10:e0005173.

3. Brady O, Johansson M, Guerra C, Bhatt S, Golding N, Pigott D, et al. Modelling adult *Aedes aegypti* and *Aedes albopictus* survival at different temperatures in laboratory and field settings. Parasites & Vectors. 2013;6:351.

4. Braks MAH, Juliano SA, Lounibos LP. Superior reproductive success on human blood without sugar is not limited to highly anthropophilic mosquito species. Medical and Veterinary Entomology. 2006;20:53-9.

5. Couret J, Benedict M. A meta-analysis of the factors influencing development rate variation in *Aedes aegypti* (Diptera: Culicidae). BMC Ecology. 2014;14:3.
