# Supplementary figures and images for: Contrasting the value of targeted versus area-wide mosquito control scenarios to limit arbovirus transmission with human mobility patterns based on different tropical urban population centers
Source: PLoS Negl Trop Dis. 2019 Jul 3;13(7):e0007479. doi: 10.1371/journal.pntd.0007479 (PMC6608929; doi:10.1371/journal.pntd.0007479)

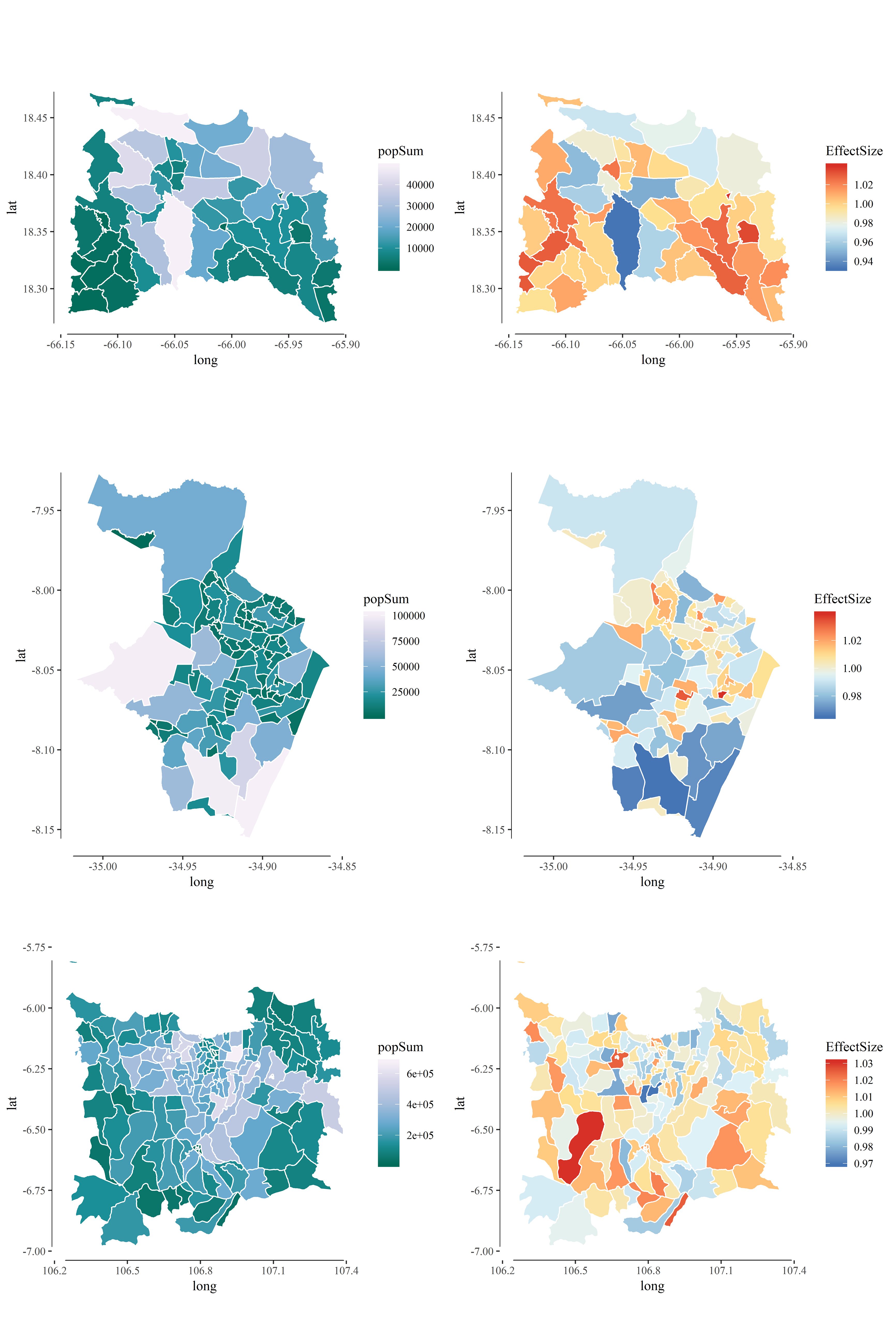

Supplement: S1 Fig — Individual neighborhoods in the San Juan area (top row), Recife (middle row) and Jakarta (bottom row), with total population size of humans per neighborhood (left) and the relative impact or effect size of including a particular neighborhood in the control response (i.e., mean prevalence of simulations in which a particular neighborhood was included divided by the mean prevalence over all simulations). The latter is based on the individual response scenario. (TIF) [file pntd.0007479.s003.tif]
